# Engaging Unmotivated Smokers to Move Toward Quitting: Design of Motivational Interviewing–Based Chatbot Through Iterative Interactions

**DOI:** 10.2196/20251

**Published:** 2020-11-03

**Authors:** Fahad Almusharraf, Jonathan Rose, Peter Selby

**Affiliations:** 1 The Edward S. Rogers Sr. Department of Electrical & Computer Engineering Faculty of Applied Science & Engineering University of Toronto Toronto, ON Canada; 2 Nicotine Dependence Clinic Centre for Addiction and Mental Health (CAMH) Toronto, ON Canada; 3 Dalla Lana School of Public Health Department of Family and Community Medicine University of Toronto Toronto, ON Canada

**Keywords:** smoking cessation, motivational interviewing, chatbot, natural language processing

## Abstract

**Background:**

At any given time, most smokers in a population are ambivalent with no motivation to quit. Motivational interviewing (MI) is an evidence-based technique that aims to elicit change in ambivalent smokers. MI practitioners are scarce and expensive, and smokers are difficult to reach. Smokers are potentially reachable through the web, and if an automated chatbot could emulate an MI conversation, it could form the basis of a low-cost and scalable intervention motivating smokers to quit.

**Objective:**

The primary goal of this study is to design, train, and test an automated MI-based chatbot capable of eliciting reflection in a conversation with cigarette smokers. This study describes the process of collecting training data to improve the chatbot’s ability to generate MI-oriented responses, particularly reflections and summary statements. The secondary goal of this study is to observe the effects on participants through voluntary feedback given after completing a conversation with the chatbot.

**Methods:**

An interdisciplinary collaboration between an MI expert and experts in computer engineering and natural language processing (NLP) co-designed the conversation and algorithms underlying the chatbot. A sample of 121 adult cigarette smokers in 11 successive groups were recruited from a web-based platform for a single-arm prospective iterative design study. The chatbot was designed to stimulate reflections on the pros and cons of smoking using MI’s running head start technique. Participants were also asked to confirm the chatbot’s classification of their free-form responses to measure the classification accuracy of the underlying NLP models. Each group provided responses that were used to train the chatbot for the next group.

**Results:**

A total of 6568 responses from 121 participants in 11 successive groups over 14 weeks were received. From these responses, we were able to isolate 21 unique reasons for and against smoking and the relative frequency of each. The gradual collection of responses as inputs and smoking reasons as labels over the 11 iterations improved the F1 score of the classification within the chatbot from 0.63 in the first group to 0.82 in the final group. The mean time spent by each participant interacting with the chatbot was 21.3 (SD 14.0) min (minimum 6.4 and maximum 89.2). We also found that 34.7% (42/121) of participants enjoyed the interaction with the chatbot, and 8.3% (10/121) of participants noted explicit smoking cessation benefits from the conversation in voluntary feedback that did not solicit this explicitly.

**Conclusions:**

Recruiting ambivalent smokers through the web is a viable method to train a chatbot to increase accuracy in reflection and summary statements, the building blocks of MI. A new set of 21 *smoking reasons* (both for and against) has been identified. Initial feedback from smokers on the experience shows promise toward using it in an intervention.

## Introduction

### Background

Cigarette smoking contributes to more than 6 million annual preventable deaths worldwide [1]. Canadians face the same threat, with 4.6 million Canadians smoking cigarettes in 2017 [2]. Although there are many evidence-based smoking cessation interventions, including behavioral counseling and medication [3], these are only effective in motivated smokers. The majority of smokers, however, are ambivalent about smoking and are not actively seeking help [4,5]. A key step is to motivate smokers to seek help, which has traditionally been done by clinicians and therapists during clinical encounters. Motivational interviewing (MI) [6] has been shown to be an effective approach.

MI is a patient-centered collaborative counseling method that attends to the language of change as a way to resolve the common problem of ambivalence [6]. A trained MI therapist uses 4 processes: engaging the patient, focusing on what matters to the patient to identify a potential target for behavior change, eliciting patients’ motivations to change, and planning concrete steps to achieve the desired outcomes. The underlying spirits of MI are compassion, acceptance, partnership, and evocation, rather than directing the patients toward solutions. A practitioner of MI listens for preparatory *change talk* (desires, abilities, reasons, and needs to change) and uses open-ended questions, affirmations, reflections, and summary statements to elicit a commitment to change and preparation to change. The practitioner identifies steps already taken that can be built upon or amplified to achieve the desired outcome. MI has been shown to achieve a small-to-medium effect size across a variety of health behaviors, including smoking cessation [7,8].

One barrier to MI-based therapy is the time it takes for therapists to learn this modality, to gain mastery, and to be consistent in their responses over time with their clients. Besides, there are not enough health care professionals to provide such interventions in person to all smokers who might benefit from MI. If a computer-based conversational agent (also known as a chatbot) could successfully employ MI techniques, it would be easy to scale the access of many smokers to this known successful intervention. It would be both low cost and much easier to access through the many chat platforms available today. However, there are substantial barriers to overcome to create such a conversational agent.

### Conversational Systems

Until recently, it has been difficult for computers to comprehend and respond appropriately to free-form text answers. This capability would allow for reflection and summary statements necessary to the key *change talk* goal of MI. Recent advances in the natural language processing (NLP) field have made it feasible to classify free-form answers into categories, which can enable a computer to select from appropriate, category-specific answers [9]. NLP focuses on the extraction and processing of specified or implied information in linguistic expressions. Conversational systems receive text or speech utterances from humans as input and generate one or more responses as output. Longer conversation chatbots (such as Microsoft’s social chatbot Xiaolce [10,11]) require 3 capabilities: the ability to extract meaning out of utterances, the ability to maintain the conversation context, and the ability to generate appropriate responses [12].

Conversational systems typically use 1 of the 2 approaches to obtain information from linguistic expressions: a rule-based approach (such as the well-known 1960s-era ELIZA chatbot [13]) and a probabilistic approach [14]. In a rule-based approach, the processing is based on rules defined by expert knowledge in a specific domain. An embodiment of the rule might be the creation of a conversation *tree*, which dictates what question to ask after a specific answer is given. In contrast, the probabilistic NLP approach learns how to classify answers and possibly generate responses from a corpus of training text that illustrates many examples of related conversations.

Modern chatbots use a combination of rule-based and probabilistic approaches—the natural language understanding (NLU) models inform the rules of the conversation context and response generation. Moreover, chatbots are increasingly being used for mental health using *chat* platforms, such as Messenger (Facebook), WhatsApp (Facebook), and WeChat (Tencent) [15-17]. For example, Woebot [18,19] helps individuals with anxiety and depression using cognitive behavioral therapy and was shown to be effective in treating depression. ElizzBot is available for consulting family caregivers [20]. Although these systems are available for people already seeking help, there are few previous studies on automated chatbots that address the step of motivating individuals to seek behavior change.

### Objectives

The long-term goal of this study is to create a chatbot that helps smokers move toward the decision to quit smoking. If such a chatbot is effective, it would be very simple and low cost to deploy to interact with a large set of unmotivated-to-quit smokers. This can be done through the platforms mentioned earlier or recruitment advertisements through social media channels. This study describes the first step in the creation of such an agent: a single-arm prospective study used to refine the responses of the chatbot and report on the experience of subjects on engaging the chatbot in an automated conversation about cigarette smoking.

## Methods

The first step was to design the structure of a minimal conversational agent that was both automated and employed the principles of MI. This was evolved through discussion and interaction between the MI expert and clinician, and the computer engineering and NLP experts. The MI behavior change approach is to engage in a conversation that causes self-reflection with the goal of reducing smokers’ ambivalence toward quitting smoking [21]. The first key decision was to have the agent prompt subjects to articulate both the pros and cons of smoking, as they are discussed in detail in the *Conversation Structure* section. A key feature identified was the need for the chatbot to provide a nonjudgmental conversation by reflecting responses, summarizing them, and then inviting further reflection. This prototype chatbot was then tested on a sample of smokers as described in the *Recruitment of Subjects* section and iterated on to gather training data to augment the set of pro and con categories the chatbot can correctly classify. The responses and labels given by subjects over the iterations were used to train and improve the NLU classifiers employed by the chatbot.

### Recruitment of Subjects

Subjects were recruited through the web from the Prolific platform [22], a website that allows researchers to offer individuals the opportunity to participate in human research experiments in exchange for financial compensation. Prolific allows researchers to select specific features from a large demographic of more than 60,000 individuals. Many of the participants reside in the United Kingdom where the company is based, but there is a significant number of participants from around the world. Textbox 1 lists the inclusion and exclusion criteria used in the subject recruitment. The *prolific rating* in Textbox 1 is the percentage of studies for which the participant has been approved in prior studies, meaning that the person running the experiment agreed that the participant’s work was acceptable. It is also worth noting that there were no criteria related to a subject’s motivation to quit, as this phase of the research aims to collect data from subjects with different motivations toward quitting.

**Inclusion criteria and exclusion criteria.**

Inclusion criteria:

- English speaker
- Smokes cigarettes daily
- Resides in the United Kingdom, the United States, or Canada
- Aged between 16 and 60 years

Exclusion criterion:

- Prolific rating <9

### Procedure

Subjects were presented with a consent form on the Prolific website, detailing the study’s information and asking them to converse with the chatbot for the purpose of training it. With consent, subjects were then asked to visit the website, where the chatbot is deployed, and complete 2 tasks: first, to converse with the chatbot, and second, to give feedback on the overall experience of conversing with the chatbot and to make suggestions for improvement. The latter was prompted by the following question at the end of their engagement with the chatbot: “Before you finish the study, please take some time to comment on your experience chatting with the chatbot. Other than its ability to understand more of your responses, what kind of advice can you give us to improve it?”

Participants were recruited in groups of 10 or 11 participants at a time. This allowed us to retrain the chatbot in between groups and improve its classification accuracy for the next group. It also enabled the incremental addition of new categories for and against smoking, which form the central part of the conversation, as described in the *Conversation Structure* section. After each group was recruited, we determined whether the training was sufficient or if more groups were needed based on the number of new distinct categories being discovered and the accuracy of the classification achieved by the smoking reasons classifier.

### Conversation Structure

**Figure 1:**
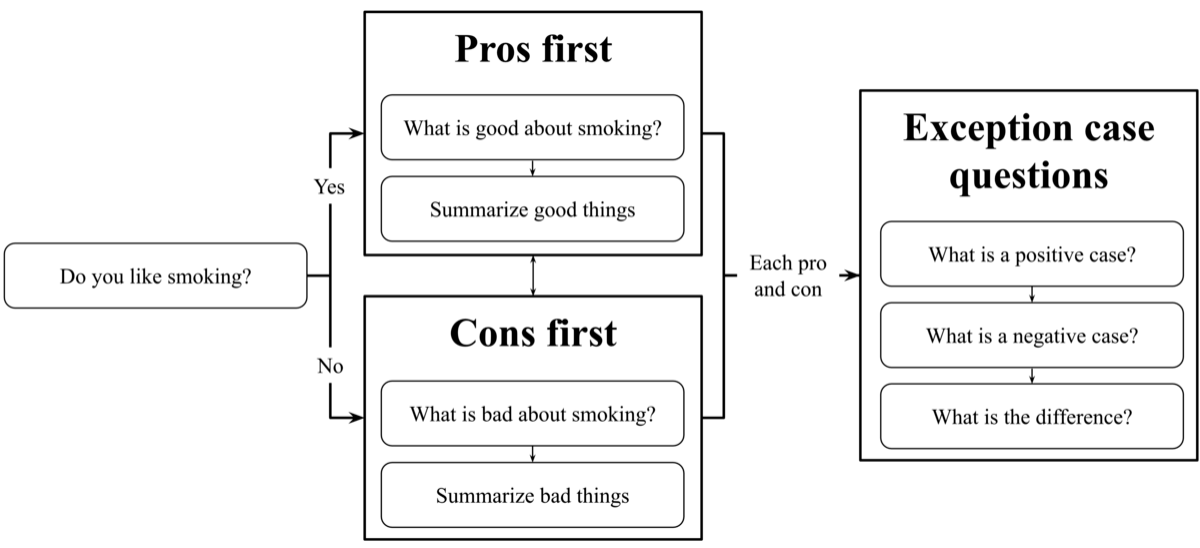
Overall flow of reflection phase of chatbot.

The chatbot delivers the conversation to subjects in 3 stages: introduction, reflection, and ending. During the introduction stage, the chatbot describes its purpose to subjects and asks for permission to continue the conversation. The reflection stage is the core of the conversation. It asks most of the questions with the goal of eliciting reflective statements about smoking behavior. To establish this reflection, the chatbot engages subjects in 2 exercises. First, it uses the MI running head start technique [21] by asking subjects to give reasons they have for smoking (called *pros*) and reasons they have against smoking (called *cons*). We also referred to these pros and cons as *smoking* *reasons*. Second, it follows this with a set of questions specifically adapted to each pro or con, which stimulate subjects to contemplate the influence of each pro and con on their behavior. Figure 1 illustrates the overall flow of the key parts of the conversation in the reflection stage. The specifics shown in Figure 1 are explained in detail in the following sections.

### Response Generation

In general, responses are selected based on the current state of the conversation and the chatbot’s classification of subjects’ responses. There are 2 types of scripted responses that the chatbot can generate. The first type includes verbatim responses in expectation of a certain situation in the conversation tree. For example, the chatbot always asks subjects, “Do you like to smoke?” when it begins the reflection stage of the conversation. The second type is a scripted response where the chatbot can generate responses that contain variables taken from conversing with the subject. For example, the chatbot can generate the following variable response: “You said ‘...’, which I believe can be classified as ‘...’.” The first ellipsis in the response is a sentence the chatbot is recalling, and the following ellipse is its understanding of the sentence. In both types of responses, the chatbot has different variations of sentences that it can choose from to reduce repeating itself in the conversation. At any point in response generation, the chatbot chooses randomly from the set of available verbatim or variable responses.

### Running Head Start

After the introduction stage of the conversation, the chatbot begins by deploying the running head start technique, which explicitly asks subjects for their pros and cons, for and against smoking. This approach provides a concrete basis for discussion, and it has been used in MI as a way to kick-start change talk, which is the eventual goal of the study [21].

Subjects are asked the following 3 questions: (1) Do you enjoy smoking? (2) What is good about smoking? and (3) What is bad about smoking? The answer to the first question determines the subject’s sentiment toward smoking and is used to order the 2 subsequent questions. If the subject’s response to the first question is positive, then the subject is asked, “What is good about smoking?” first. Similarly, if the response is negative, then the subject is asked, “What is bad about smoking?” first. This ordering aligns with the MI principle of keeping the conversation open ended and client centered [21]. The purpose of the second and third questions is to accumulate the subject’s pros and cons of smoking to pursue the key MI concepts of reflection and summarizing [21].

When responding to requests for the pros and cons of smoking, the subject provides free-form textual responses. NLU requires a corpus of training data that contains examples of the free-form responses together with labels that classify the response into a relevant category. A key aspect of this training phase of the chatbot is to determine the name and quantity of distinct categories of pros and cons—the smoking reasons—that would exist in all ambivalent smokers. Once these categories are established, we will be able to provide tailored responses to the subjects during an intervention study, which is discussed at the end of this paper.

The chatbot provides an initial response to each pro or con given by the subject, which reflects a classification (ie, categorization) of their pro or con. This mimics the general MI approach to communicate understanding by the MI practitioner to their clients. For example, when a subject says, “Smoking gives me a pleasurable and happy feelings,” the chatbot replies with, “I understand, you get pleasure and satisfaction from smoking.” This confirmatory reply reflects an apparent understanding of the chatbot. Reflection is most useful to subjects when they write long utterances that can be distilled into smaller ones or when they express multiple pros or cons that fall under the same category [21].

Once the subject has finished providing all of their pros and cons, the chatbot summarizes by repeating everything the subject has written, verbatim, within each category. For example, the chatbot might write, “You said ‘the way it makes me feel’, which I believe is essentially feeling good or satisfaction.” In addition, the chatbot responds correctly if subjects write multiple pros or cons that are classified into the same category. It does that by summarizing each category in one utterance. For example, the chatbot summarizes 2 pros as pros in the stress category by saying, “You said ‘calms nerves’, and ‘relieves stress’, which I think are related to stress.” To show how the chatbot applies the running head start technique with subjects, Textbox 2 provides an example taken from a conversation with a recruited subject.

In summary, this first exercise of the reflection stage in the design of the conversation follows several MI principles: it aligns with the subject’s sentiment toward smoking to determine whether pros or cons should be discussed first. Second, the conversation explores the pros and cons, reflecting each one in a shorter form. Finally, the chatbot brings all the pros and cons together to summarize at an appropriate point in the conversation. The *Exception Case Questions* section describes specific follow-up questions for each summarized pro and con that were given by the subject.

**Example of reflection and summarization in the conversation.**

Conversation example:

- Bot: “Do you like to smoke?”
- Subject: “I do”
- Bot: “What do you enjoy about smoking?”
- Subject: “It is a great way to kill some time between tasks”
- Bot: “I get it, smoking is helpful because it provides a useful distraction for you”
- Bot: “What else do you enjoy about smoking?”
- Subject: “it is relaxing when doing somehthing stressful”
- Bot: “Let me see if I understand: smoking relieves you of stress”
- Bot: “You said ‘It is a great way to kill some time between tasks,’ which I think is related to distraction”
- Bot: “You also said ‘it is relaxing when doing something stressful,’ which I believe can be classified as stress”
- Bot: “Did I summarize everything back correctly?”
- Bot: “Okay. Let’s now chat about the bad things about smoking”

### Exception Case Questions

One of the biggest determinants that predict whether someone seeking change will take action is self-efficacy [23], which can be summarized as a person’s belief in their own competence to achieve the change. To stimulate reflection toward this end, the chatbot invites the person to reflect on the reasons they gave for and against smoking. There are 2 desired outcomes to this approach: first, to have subjects enhance their own understanding of how a specific pro or con leads them to smoke or not smoke, and second, to identify situations where subjects were able to refrain from smoking and to reflect on how and why that was possible and how they might *generalize* from those situations and be able to resist the urge to smoke more often.

To achieve these outcomes, the chatbot enquires about exceptions to the subject’s usual behavior. For each given pro or con, the chatbot asks 3 questions. The first question asks the subject to recall a situation in which they experienced the pro or con and how it led them to smoke (called the *positive* case). The second question asks them to remember a situation in which they experienced the same pro or con but did not end up smoking (called the *negative* case). The third question asks the subject to identify the difference between the positive and negative cases by reflecting on what might have caused the difference in outcome. These questions are designed to have the subject contemplate what triggers them to smoke and elicit examples when they successfully deferred or avoided smoking. These questions are consistent with the MI approach using a guiding form of communication rather than a directive form [21]. An example of the 3 questions taken from our training data collection is shown in Textbox 3.

**An example of the 3 exception case questions.**

Bot: “Please describe a time when you experienced stress and ended up smoking.”

Subject: “Smoking is associated with ‘de-stressing’ for me. If I feel anxious smoking takes the edge off.”

Bot: “Please describe a time when you experienced stress and didn’t end up smoking”

Subject: “Before I got divorced my ex-husband used to really stress me out but he also made me feel bad about smoking. Many times he would be stressing me out and I would not choose to smoke around him.”

Bot: “Think back to the time when you were able to prevent yourself from smoking. What made it different from when you did smoke?”

Subject: “The emotional baggage involved with having that cigarette far out weighed any pleasure I would gain from smoking it, so I didn’t do it.”

### Smoking Reason Categories and Iterative Improvement

Before the first training iteration, it was necessary to have an initial set of smoking reasons (pro and con) categories so that the NLU model can attempt to classify them to inform the chatbot how to respond. The original set of 10 reasons was taken from the following 2 sources: the first 6 reasons came from the “Why do I Smoke” questionnaire [24]: pleasure, stimulation, stress, routine, craving, or “having something in their hand.” An additional 4 reasons were deemed common reasons for smoking by a smoking cessation and MI expert: addiction, health, cost, and cool.

Although these 10 categories generally indicate pro smoking reasons, some of them could also be considered as a reason against smoking. For example, a con in the stress category is smokers’ feeling of anxiety when they run out of cigarettes. That is something bad about smoking. Thus, for each category, we also determined if a reason was a pro, a con, or both. This was important to detect, as the reflection and summarization responses selected by the chatbot are different depending on whether the reason is a pro or a con.

In addition to the abovementioned 10 categories, in the training study, the chatbot asks subjects to determine if the classification selected by the chatbot is correct, and if not, to give the correct category, from a list. In the case that the subject believes that a list of categories does not include the reason, the chatbot asks subjects to suggest a new category. We refer to this category confirmation process as the *training mode* of the chatbot and is illustrated with 2 examples in Textbox 4.

**Chatbot training mode.**

Bot: “Do you have more positive things about smoking? Tell me if you can think of any”

Subject: “Helps me to bond with other smokers and socialize”

Bot: “I understood ‘Helps me to bond with other smokers and socialize’ as something related to ‘Socializing’”

Bot: “Am I right?”

Subject: “Right”

Bot: “Thank you for confirming my knowledge”

Bot: “I understand, smoking is a social habit for you”

Bot: “What else do you find positive about smoking?”

Subject: “I like the way the smoke tastes”

Bot: “I understood ‘I like the way the smoke tastes’ as something related to ‘Feeling good’”

Bot: “Am I right?”

Subject: “Wrong”

Bot: “Is it on of the following options? Addiction, Stimulation, ... , Other”

Subject: “Other”

Bot: “Can you please tell me what it is in one or two words?”

Subject: “Flavor”

Bot: “Thank you for teaching me something new!”

Bot: “What else do you dislike about smoking?”

Subject: “yellow fingers”

Bot: “I understood ‘yellow fingers’ as something related to ‘Health’”

Bot: “Am I right?”

Subject: “Wrong”

Bot: “Is it on of the following options? Addiction, Stimulation, ... Physical Appearance, ..., Other”

Subject: “Physical Appearance”

After each group of subjects finished their respective conversation, new information about the smoking reason categories emerged and required consideration by the researchers. This information can be one of the following, as shown in Textbox 4: (1) confirmation of category detection, (2) correction of category detection, and (3) introduction of a new category. To ensure the validity and consistency of the category information provided by subjects, we reviewed each confirmation, correction, and introduction after each training group and before retraining the chatbot for the next one. In our review, we validate that confirmations and corrections are indeed valid and are not the results of typing or subject error.

We also considered the introduction of new categories by reviewing the conversation and subject responses that suggested them. If, on discussion, we could not reach consensus that a response fit into an existing category, then a new category was created and given a descriptive name. As a result, we need to create scripted responses so that the chatbot can reflect and summarize the new categories on detection. The process for scripting new responses is similar to how we agree on a new category; it is decided based on discussion among the researchers. As we follow an iterative strategy in engaging subjects and training the chatbot, more pro and con categories appeared, as subjects introduced new reasons to the chatbot through this training mode in the conversation.

### Analysis

The primary goal of the study is to build a data set of example pro and con reasons for smoking and their categories. The secondary goal is to make the conversation experience with the chatbot as pleasant as possible. To evaluate these goals, we counted the number of examples of the pros and cons and their categories. We also calculated the precision, recall, and F1 score of the chatbot’s classifiers on a set of examples that it has not been trained on to measure its performance. Precision measures the percentage of correct detections from all the detections a classifier makes on the test data, whereas recall measures how often the classifier incorrectly misclassified responses or was unable to determine any classification. The F1 score is the harmonic mean of precision and recall, and it measures the performance of the classifier in its ability to generate the correct detections (precision) and not miss any of them (recall). Table 1 lists the 3 measurements used for evaluating the 2 classifiers. All 3 metrics are calculated as micrometrics: true positive (TP), false positive (FP), and false negative (FN) are calculated globally across the classifier classes.

**Table 1:** Definitions of precision, recall, and F1 score.

Measurement	Definition
Precision	TP^a^ (TP + FP^b^)
Recall	TP / (TP + FN^c^)
F1 score	TP / (TP + 0.5 x [FP + FN])

^a^TP: true positive.

^b^FP: false positive.

^c^FN: false negative.

When calculating the metrics in Table 1, a TP event is defined as the event when the chatbot can correctly detect the presence of a category in the subject’s utterance and generate an appropriate response. An FP event is observed when the chatbot generates an incorrect classification and provides an off-target response to a subject’s utterance. Finally, an FN is observed when the chatbot is unable to generate any classification on a given subject’s utterance.

### Ethics Review

The University of Toronto Health Science Research Ethics Board (REB) reviewed and approved the study. The REB protocol number is 35962 and was approved on May 28, 2018.

## Results

### Participants

A total of 121 participants completed the study in 14 weeks. Table 2 gives the participants’ demographic information, including their age, sex, smoking frequency, last quit attempt, employment status, and country of residence. This information was entered by participants when they first registered with Prolific, and it is possible that not all fields have responses from all participants. Information that was not provided by participants to Prolific is marked as *missing* in Table 2.

**Table 2:** Demographics of the subjects in the study (N=121).

Characteristics		Values
Age (years), mean (SD)		35.2 (9.8)
**Age (years), n (%)**		
	16-19	0 (0.0)
	20-29	40 (32.8)
	30-39	42 (34.4)
	40-49	23 (18.9)
	50-59	13 (10.7)
	60	1 (0.8)
	Missing	3 (2.5)
**Sex, n (%)**		
	Female	60 (49.2)
	Male	59 (48.4)
	Missing	3 (2.5)
**Smoking frequency, n (%)**		
	Once a day	5 (4.1)
	2-5 times a day	20 (16.4)
	6-10 times a day	29 (23.8)
	11-19 times a day	48 (39.3)
	≥20 times a day	19 (15.6)
	Missing	1 (0.8)
**Last quit attempt, n (%)**		
	Never	16 (13.1)
	>12 months	30 (24.6)
	7-12 months	10 (8.2)
	4-6 months	14 (11.5)
	1-3 months	15 (12.3)
	Currently trying	14 (11.5)
	Missing	23 (18.8)
**Employment status, n (%)**		
	Full time	61 (50.0)
	Part time	21 (17.2)
	Not in paid work	17 (13.9)
	Unemployed (and job seeking)	15 (12.3)
	Other	7 (5.7)
	Missing	1 (0.8)
**Country of residence, n (%)**		
	United Kingdom	66 (54.1)
	United States	50 (41.0)
	Canada	6 (4.9)

### Participant Interaction

The chatbot received 6568 responses from the 121 participants, where 4271 were free-form responses and 2297 were selected from the suggested responses from the chatbot. The mean time spent by each participant interacting with the chatbot was 21.3 (SD 14.0) min. The shortest conversation was 6.4 min long, whereas the longest conversation lasted for 89.2 min. The longest conversation was a result of a specific participant having more pros and cons about smoking as well as crafting long responses to the chatbot.

### Smoking Reasons Data Set

An additional 11 distinct categories of smoking reasons separate from the original 10 categories were identified. Table 3 provides the entire list and indicates whether a category can be considered as a pro, con, or both. It also provides an example statement from a study participant in each category.

This study produced a data set of 1010 samples of how participants expressed 21 distinct categories of why smoking is good or bad. Of these 1010 samples, 79 samples and 10 categories were synthesized by the researchers before the initial group of participants, as described earlier. The remaining 931 samples and 11 categories were generated through the input of the recruited participants. Of the 1010 samples, 490 expressed pros about smoking and 520 expressed cons about smoking. Of the 21 distinct categories, 5 are pro categories (boredom, cool, feel good, something in my hand, and stimulation), 6 are con (cost, dirty, fire hazard, physical appearance, poor role model, and stigma), and the remaining 10 are both pro and con (addiction, distraction, flavor, focus, health, routine, smell, smoking restriction, social, and stress). Table 3 also lists the number of responses acquired in each reason category.

**Table 3:** A list of all the smoking reason categories used in the conversation.

Category and pro or con			Count		Example
**Addiction**					
	Con	1		“The first thing I think of when I wake up is my first cigarette”	
	Pro	55		“I crave nicotine”	
**Boredom**					
	Pro	16		“I smoke out of boredom”	
**Cool**					
	Pro	9		“Makes me look cool”	
**Cost**					
	Con	67		“Cost so much for such little joy”	
**Dirty**					
	Con	16		“The cigarette ash drops about”	
**Distraction**					
	Pro	52		“It gives me time to myself and time to think”	
	Con	11		“how it interrupts your work”	
**Feel good**					
	Pro	85		“I enjoy the feeling that it gives me”	
**Fire hazard**					
	Con	7		“It burns my home and furniture”	
**Flavor**					
	Pro	25		“I like the way the smoke tastes”	
	Con	7		“The taste smoking leaves in your mouth”	
**Focus**					
	Pro	15		“Helps me concentrate doing computer work”	
	Con	3		“Can’t concentrate if I need a cigarette”	
**Health**					
	Pro	25		“Cigarettes help with bowel movements”	
	Con	133		“All the health problems smoking is linked to causing”	
**In my hand**					
	Pro	30		“Gives me something to occupy my hands with”	
**Physical appearance**					
	Con	31		“Aging or appearance change is always a fear”	
**Poor role model**					
	Con	14		“I feel guilty because my son doesn’t like me smoking and nags”	
**Routine**					
	Pro	9		“It something to look forward to doing”	
	Con	5		“routine cigarettes”	
**Smell**					
	Pro	9		“The smell of cigarette smoke is nice”	
	Con	74		“Leave you smelling on fingers breath and clothes”	
**Smoking restriction**					
	Pro	2		“Sometimes smoking restrictions will force me to go outside and then I realize it’s a beautiful night and i’m glad it forced me to go outside”	
	Con	22		“You can’t smoke in a lot of places”	
**Social**					
	Pro	58		“I like to socialise with other people who smoke”	
	Con	11		“people around me do not smoke, only I do”	
**Stigma**					
	Con	62		“Being made to feel unwelcome by non smokers”	
**Stimulation**					
	Pro	37		“The sensation of the tobacco as it catches my throat”	
**Stress**					
	Pro	177		“Helps me relax and decrease stress”	
	Con	2		“Feeling anxious when I’ve run out”	

### Classifier Training Result

In implementing the software for the chatbot, we originally used a third-party web-based system for classification called Wit.ai [25], referred to as classifier SR1 (smoking reasons [classifier] 1). Although it made the ramp-up of the classification easier, we did not have the ability to understand and control the behavior of the *black box* classifier. This led us to build our own classifier using the NLP framework from spaCy [26], referred to as classifier SR2 (smoking reasons [classifier] 2). Using the entire data set obtained from the 121 participants (810/1010, 81.2% used as training data and 200/1010, 19.8% used as test data), we measured the performance of the 2 classifiers. Table 4 gives the overall capability of the 2 classifiers used after all the training data were collected. Although classifier SR1 has slightly better precision than SR2, the latter has a much better recall and thus gives a much better overall F1 score. Recall can informally be thought of as the chatbot’s ability to correctly reflect what subjects are expressing. Higher degrees of recall means that the chatbot is able to detect more of the subjects’ pros and cons and generate reflections on them.

**Table 4:** Precision, recall, and F1 score for the smoking reasons classifier 1 and smoking reasons classifier 2 natural language understanding classifiers.

Measurement	Smoking reasons classifier 1	Smoking reasons classifier 2
Precision	0.98	0.87
Recall	0.28	0.84
F1 score	0.44	0.86

A measure of the progress of the classifiers over the training group iterations is given in Table 5, which shows the precision, recall, and F1 score that each participant group experienced during the actual training session. (This is quite distinct from the results in Table 4 because those are given when the classifier is trained and evaluated using the entire corpus of utterances and labels.) Several things are changing as these results were acquired over time—the number of categories is increasing and the amount of training data available to train the classifier is increasing. In addition, as described, the classifier used from groups 1 to 10 was SR1, whereas group 11 used classifier SR2. Table 5 illustrates the progress of the classifier used during training, ending with a significantly better F1 score overall there as well, with classifier SR2.

**Table 5:** Precision, recall, and F1 score of the chatbot’s pro and con detection.

Group	Precision	Recall	F1 score	Classifier used
1	0.93	0.48	0.63	SR1^a^
2	0.97	0.56	0.71	SR1
3	0.96	0.26	0.41	SR1
4	0.96	0.46	0.62	SR1
5	0.93	0.67	0.78	SR1
6	0.92	0.63	0.75	SR1
7	1.00	0.61	0.76	SR1
8	0.96	0.68	0.80	SR1
9	0.93	0.58	0.71	SR1
10	0.95	0.60	0.73	SR1
11	0.91	0.75	0.82	SR2^b^

^a^SR1: smoking reasons classifier 1.

^b^SR2: smoking reasons classifier 2.

### Voluntary Free-Form Feedback

Participants were asked to voluntarily answer the following question at the end of their engagement with the chatbot: “Before you finish the study, please take some time to comment on your experience chatting with the chatbot. Other than its ability to understand more of your responses, what kind of advice can you give us to improve it?” The majority of answers to this question suggested improvements to the chatbot. However, there were other signals in the feedback that emerged. The other responses included that participants enjoyed conversing with the chatbot, found it beneficial, or were frustrated by it. To measure these data quantitatively and based on discussion among the researchers, the following 4 mutually inclusive (ie, overlapping) labels were added to each participant’s feedback: improvement suggestions, enjoyment, benefit, and frustration. Textbox 5 describes each of the 4 labels. In the study, 76.9% (93/121) of participants answered the feedback questions. The following percentages of the 4 labels were observed in their feedback: 44.6% (54/121) expressed improvement suggestions, 34.7% (42/121) expressed enjoyment, 8.3% (10/121) indicated benefit, and 2.5% (3/121) expressed frustration. Textbox 6 provides some examples of the benefit and frustration feedback received from the participants in the study.

**Description of feedback labels.**

Improvement suggestions

- Any feedback that suggested bug fixes, new capabilities, or comments relating to functionality

Enjoyment

- Feedback that indicates a positive, pleasant experience with the chatbot

Benefit

- Feedback explicitly indicating that the chatbot caused participants to reflect on their behavior or motivated them to quit

Frustration

- Any feedback that indicates the subject had a negative experience or caused a negative effect

**Feedback samples from the study.**

Benefit

- “The study actually made me think more about quitting smoking”

Benefit

- “That was actually really very helpful. It was getting my thoughts out. I have been smoking for a long time and this was the first time believe it or not that I actually got some insight to my behavior, LOVED IT.”

Frustration

- “This chatbot really needs some redesign. Punctuation seems to throw it off ‘Yes’ is accepted but ‘Yes.’ is not. Also, it’s making assumptions of people which is going to make them combative, like me, when it just goes ‘obviously you aren’t able to stop yourself ever’.”

Frustration

- “Frustrating, the questions made little sense. I had ‘it smells bad’ and it asked me to describe a time when the action ‘it smells bad’ caused you to smoke. A lot of self reflections kind of felt pointless as well. I smoke a pack a day, often without thinking about it., so pinpointing a time when something caused me to smoke is really hard.”

The free-form feedback from participants gives an informative view of their experience: almost 35% (42/121, 34.7%) of participants found conversing with the chatbot a pleasant experience. In addition, more subjects expressed benefit (42/121, 8.3%) than frustration (3/121, 2.5%). We deem the frustration effects as not harmful in the long term because the target audience are unmotivated subjects, and continuing to smoke does not have a short-term harmful effect.

## Discussion

### Principal Findings

Iteratively recruiting participants to collect training data as well as engaging them in a conversation about smoking enabled the training and validation of the MI-based chatbot. Although our chatbot does not strictly follow the 4 processes of MI mentioned in the *Introduction* section, it does follow the spirited principles of MI. It keeps the conversation client centric by tailoring the running head start technique to subjects’ sentiment toward smoking. It provides reflection and summarization to subjects’ expressed pros and cons of smoking. Finally, it uses the exception case questions to each pro and con, possibly revealing situations that might resolve ambivalence. In addition, we were able to improve the performance of our chatbot and improve its ability to hold a conversation with a relatively small number of participants. Interesting information emerged from the voluntary feedback question asked at the end of the experiment. More than 1 in 3 participants enjoyed conversing with the chatbot, and the qualitative data indicated that although a small subset of participants found benefit in engaging with the chatbot, more refinements are necessary to minimize the frustration that could affect engagement in behavior change.

In the focus area of this study, there has been some prior work on smoking cessation–based chatbots. Perski et al [27] explored adding a chatbot to a preexisting smoking cessation support mobile app with a randomized control trial. Their intervention used several of the techniques we employed in this study, including identifying reasons for wanting and not wanting to stop smoking and tailoring the interactions appropriately. They showed that using a chatbot in their mobile app increased engagement and the odds of quitting success. However, they did not make use of reflective listening, which is one of the core capabilities of our chatbot.

A key part of our designed conversation uses reflective listening in the elicitation of the pros and cons of smoking and their subsequent short-form reflection and restatement. Although our primary goal was to collect training data and test our chatbot on recruited participants, we have gained insights into how to improve our chatbot for future interventions. Our experience showed that subjects generally do not articulate short descriptions of the pros and cons of smoking. Rather, they explain feelings associated with a given pro or con. For example, a subject might say, “Helps me bond with other smokers” to communicate *socializing* as a pro for smoking. Therefore, the system must have a reasonably complete set of categories for expressing the pros and cons of smoking, such as *socializing*. The name of those categories must be reflective of the many ways that subjects may express a pro or con so that a subject can agree (or disagree) correctly when the chatbot makes a classification during the training study. In addition, the reflection responses given by the chatbot are tuned to each category; thus, choosing the right number of distinct categories, so that these responses are effective, is important.

It is worth noting that in the context of this chatbot, precision is not as important as recall. High recall is desired for providing reflections to subjects. The current implementation of the NLU classifier for detecting the pros and cons is a mutually exclusive classifier. Any given utterance expressed by the subject for a pro or con for smoking only maps to one category. However, the categories of the pros and cons do partially overlap and are subjective. For example, our chatbot classifies the utterance “how it interrupts your work” into the *focus* category instead of the *distraction* category. As a result of this classification, the chatbot will respond with “I understand, smoking makes you lose focus” instead of “I get it, smoking can be a distraction for you.” In this case, the generated response from the chatbot will most likely satisfy the subject, as it would for other categories that are close in meaning.

According to Pereira and Diaz [28], most health-related chatbots focus on neurological and nutritional disorders whereas smoking, which falls under the category of addiction disorders, is one of the less focused-on health problems tackled by chatbots. In addition, *consumability* (a description of users’ end-to-end experience with technology) and personalization are the 2 main enablers of chatbots in the health space. In this research, we also focus on these enablers to bring chatbots to the underserviced health problem of cigarette smoking addiction. Our chatbot achieves personalization by tailoring the conversation around subjects’ perspectives about the positive and negative aspects of smoking during optimization for a human-like conversation experience.

Finally, it was promising to observe that some participants gave extensive answers to the questions posed by the chatbot, and these answers were thoughtful and reflective. This suggests that when deployed as an intervention, there may be a good effect for some future participants.

### Limitations and Future Improvements

This study has 4 main limitations. The first limitation is using the running head start technique. Although this technique is recommended when there is an observed absence of *change talk* [21], we are using it to create a simple basis for discussion and reflection. We recognize that for subjects who are already exhibiting change behavior, this might not be appropriate or helpful, and in the future, we plan to improve the chatbot to detect change behavior early in the conversation and employ a different strategy.

The second limitation is the assumption that subjects are unmotivated to quit; however, we know from the demographics in Table 1 that some of the subjects may be currently trying or recently tried quitting. As the chatbot uses the running head start MI strategy to elicit reflection in subjects, this class of subject may find the elicitation of pros and cons frustrating because they are already motivated to quit [21]. One possible solution to this problem is to screen motivated smokers using techniques such as the readiness ruler and ending the conversation with them. The chatbot could also lead them to other resources appropriate for dealing with their stage of behavior change.

The third limitation is the subjects’ self-reported demographic data on the Prolific platform [22]. The collected demographics in Table 1 might be outdated. One important example is the “When was your last quit attempt” information, which is reported once subjects join the platform. This information can be stale and not indicative of the current state of the subject. In our future work, we intend to point subjects to a survey they answer before entering a conversation with the chatbot. In this survey, we can ask subjects for more recent answers about variables that might have changed from when they joined Prolific.

The fourth limitation is that the chatbot finishes the conversation after receiving the response to the exception case questions. This lack of follow-on to the exception case questions or elsewhere in the conversation can frustrate subjects and possibly lead to negative unintended effects. Generating responses on general situational reflections in the exception case questions stage of the conversation requires general NLU response generation capabilities that are being actively researched. However, in the future, we hope to provide useful responses to these reflections and to continue the conversation productively.

### Conclusions

This study has described the design and training of a conversational agent whose purpose is to interact with ambivalent smokers to move them toward quitting smoking. The agent employs strategies from MI and makes use of the running head start technique to launch a concrete discussion. A key aspect of the design is to allow free-form text responses to questions and the use of NLU techniques to correctly categorize the free-form responses. We were able to show a method to train the NLU engine to accurately identify responses, which is then used to select an appropriate sequence of responses. A side effect of the training was to identify 21 distinct categories of reasons for and against smoking that the training subjects helped to define. The next step is to conduct a feasibility study of the now-trained intervention and to iterate on the design again to improve its effectiveness.

## Abbreviations

FN: false negative
FP: false positive
MI: motivational interviewing
NLP: natural language processing
NLU: natural language understanding
REB: Research Ethics Board
SR1: smoking reasons classifier 1
SR2: smoking reasons classifier 2
TP: true positive
